# An in-depth analysis of theoretical frameworks for the study of care coordination^1^

**DOI:** 10.5334/ijic.1068

**Published:** 2013-06-27

**Authors:** Sabine Van Houdt, Jan Heyrman, Kris Vanhaecht, Walter Sermeus, Jan De Lepeleire

**Affiliations:** Katholieke Universiteit Leuven, 3000 Leuven, Belgium; Katholieke Universiteit Leuven, 3000 Leuven, Belgium; Katholieke Universiteit Leuven, 3000 Leuven, Belgium; Western Norway Research Network on Integrated Care, Haugesund, Norway; Katholieke Universiteit Leuven, 3000 Leuven, Belgium; Katholieke Universiteit Leuven, 3000 Leuven, Belgium

**Keywords:** care coordination, organizational models (Mesh), theoretical models (Mesh), review (Mesh), coordination strategies

## Abstract

**Introduction:**

Complex chronic conditions often require long-term care from various healthcare professionals. Thus, maintaining quality care requires care coordination. Concepts for the study of care coordination require clarification to develop, study and evaluate coordination strategies. In 2007, the Agency for Healthcare Research and Quality defined care coordination and proposed five theoretical frameworks for exploring care coordination. This study aimed to update current theoretical frameworks and clarify key concepts related to care coordination.

**Methods:**

We performed a literature review to update existing theoretical frameworks. An in-depth analysis of these theoretical frameworks was conducted to formulate key concepts related to care coordination.

**Results:**

Our literature review found seven previously unidentified theoretical frameworks for studying care coordination. The in-depth analysis identified fourteen key concepts that the theoretical frameworks addressed. These were ‘external factors’, ‘structure’, ‘tasks characteristics’, ‘cultural factors’, ‘knowledge and technology’, ‘need for coordination’, ‘administrative operational processes’, ‘exchange of information’, ‘goals’, ‘roles’, ‘quality of relationship’, ‘patient outcome’, ‘team outcome’, and ‘(inter)organizational outcome’.

**Conclusion:**

These 14 interrelated key concepts provide a base to develop or choose a framework for studying care coordination. The relational coordination theory and the multi-level framework are interesting as these are the most comprehensive.

## Introduction

Current healthcare systems have evolved mainly in response to delivering acute episodic care; therefore, they are not well designed for dealing with complex chronic illnesses [1, 2]. The fact that patients with complex chronic conditions often require long-term care from different healthcare and social care professionals, both in the community and the hospital, has led to an increasing need for care coordination to ensure good quality care [3–5].

Strategies to improve care coordination are frequently developed to ensure good quality care. The fact that these strategies do not always lead to the desired outcome [6–8] is due in part to the lack of clarity about the concept of care coordination and the theoretical frameworks for evaluating interventions, and in part to the uncertainty in how best to measure care coordination [5].

The lack of clarity about the concept of care coordination is a result of multiple existing definitions and models for care coordination, each with a different emphasis. The landmark study in this domain was the review conducted for the Agency for Healthcare Research and Quality [5]. That study presented over 40 heterogeneous definitions of care coordination. To address this problem, a definition of care coordination was developed based on five key elements found in the identified definitions. Accordingly, care coordination is currently defined as “the deliberate organization of patient care activities between two or more participants (including the patient) involved in a patient’s care to facilitate the appropriate delivery of health care services. Organizing care involves the marshalling of personnel and other resources needed to carry out all required patient care activities, and is often managed by the exchange of information among participants responsible for different aspects of care” [5].

In the landmark review [5], five theoretical frameworks were described to show how theoretical thinking can enrich the study of care coordination. These included the Andersen Behavioral Model [9]; the Donabedian Quality Framework [10]; the Organizational Design Framework [11], where Wagner’s Chronic Care Model [2] was described as an example in the landmark review; the Relational Coordination Framework [12]; and the related Multi-level Framework [13]. The Andersen Behavioral Model and the Donabedian Quality Framework were both adapted in the landmark review for the study of care coordination. The search for theoretical frameworks in the landmark review was exploratory and considered as a starting point for a more systematically search.

More clarity about the key concepts related to care coordination and the relationship between these concepts is needed to study what strategies work when and how and to develop measurements for care coordination [5].

The aim of this study is two-fold: 1) to provide an updated overview of the existing theoretical frameworks for the study of care coordination with a review of the literature, and 2) to perform an in-depth analysis of all identified theoretical frameworks to clarify key concepts related to care coordination.

## Methods

The databases PubMed and ISI Web of Knowledge were searched with the following search strategy: in the title and abstract, we searched for coordinat* and ‘care’ and [(theor*) or ‘model’ or ‘framework’ or (concept*)]. The search was limited to articles published between 2007 and 2010, because our aim was to update the 2007 review of the Agency for Healthcare Research and Quality. We extended the search by examining without a date limit the references of relevant articles and searching Google and websites of quality improving organisations [14–21].

The retrieved articles were screened by examining the titles and abstracts to identify theoretical frameworks or references to theoretical frameworks that were related to care coordination. The included articles were then fully reviewed. To ensure reliability, we randomly selected one-third of the fully reviewed articles for blind review by a second researcher (JDL, WS, or JH). There were no discrepancies in the inclusion or exclusion of articles. Articles were excluded when: 1) the definition of care coordination did not correspond to any of the key elements defined in the Agency for Healthcare Research and Quality review [5]; 2) no theoretical framework was described, meaning no links or relationships between the concepts defined were established; or 3) no application of the theoretical framework was found in a healthcare setting, either in the initial search or in the Web of Knowledge search for articles that referred to theoretical frameworks. In addition, we excluded studies describing the development or implementation of coordination strategies or articles that referred to frameworks for implementing coordination strategies to focus on theoretical framework for the study of care coordination. No restrictions were imposed based on the language or type of article. The titles and abstracts of all non-English articles were available in English. Seven of these articles were fully reviewed: five were in French and two in German. An assessment of all the articles was drawn up in an Excel file. The references and the assessment decisions were tracked in Reference Manager 11.

An in-depth analysis of the retrieved theoretical frameworks was performed according to the analytic technique described by Walsh and Downe [22]. First, the original author’s understanding of the concepts in each theoretical framework was identified and tabulated. Second, these concepts were compared among all articles in terms of similarities and differences, and then they were structured into key concepts. Finally, we came to a consensus on the construction and rationale for the different key concepts.

## Results

**Figure 1.**
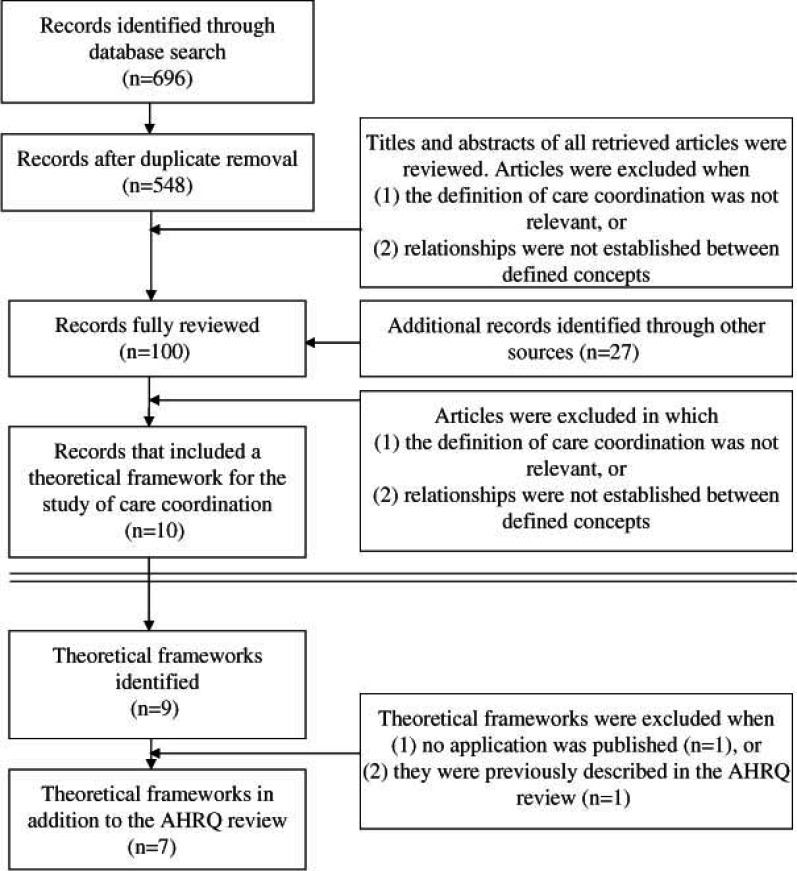
Flow chart of study selection, from search to inclusion.

We found 696 potentially relevant articles. After the removal of duplicates, 548 articles were screened on the basis of their title and abstract. Of those, 100 articles were fully reviewed. After examining the references and websites of quality improving organizations, 27 additional articles were fully reviewed. This full review identified seven theoretical frameworks in addition to the frameworks described in the 2007 Agency for Healthcare Research and Quality’s review. Figure 1 presents a flow chart of the selection process, from search to inclusion.

All the newly retrieved theoretical frameworks were developed by a research agency located in the US, except the framework of team performance that was developed by researchers in the UK (Table 1). The theoretical frameworks were published between 1991 and 2009. Four of the newly retrieved theoretical frameworks were published before 2007, but were not included in the Agency for Healthcare Research and Quality review. These additional frameworks were found via references cited in relevant articles. The focuses of the theoretical frameworks differed.

The most frequently cited theoretical framework in Web of Knowledge was the Time, Interaction, and Performance Theory. Three theoretical frameworks were not cited in Web of Knowledge; these frameworks were applied in articles identified in our initial search.

All newly retrieved theoretical frameworks (n=7) and the frameworks previously identified in the Agency for Healthcare Research and Quality review (n=5) were included in the in-depth analysis. The first step of the in-depth analysis of the identified theoretical frameworks (n=12) resulted in the formulation of 104 concepts. Next, a comparison of these concepts among the different theoretical frameworks led to the identification of 14 key concepts that these frameworks addressed (Table 2).

The first key concept of care coordination was defined as ‘external factors’. Two theoretical frameworks addressed external factors. These frameworks focused on how care coordination was affected by national health policy, economic factors, and dependency on regulations and existing resources.

The second key concept of care coordination was defined as the “structure of a team, organization, or inter-organizational network”. Seven theoretical frameworks addressed the physical and organizational aspects that support and direct the provision of care. These frameworks considered several factors that influenced the structure of a team, organization, or inter-organizational network, including:

- the number of participants
- the specializations
- the way participants were grouped
- the number of linkages between participants
- the amount of information required to manage the care of the patient or patient group
- the existing mechanisms for coordinating the care provided by the different participants; for example, leaders or structural links across the boundaries between disciplines, units, or organizations.

The third key concept of care coordination was defined as the ‘characteristics of the task’. Five theoretical frameworks addressed task characteristics that influenced care coordination including:

- the degree of variability or standardization of the tasks
- the degree to which team members depended upon each other
- the simplicity or complexity of the tasks
- the degree of certainty in the outcome

Other factors that affected care coordination were:

- the importance and length of the task
- the workload
- the time pressure

The fourth key concept of care coordination was defined as ‘cultural factors’. Only one theoretical framework incorporated cultural factors, which focused on attitudes, beliefs, norms, and values.

The fifth key concept of care coordination was defined as ‘knowledge and technology’. Six theoretical frameworks addressed factors of ‘knowledge and technology’. These frameworks focused on available skills, expertise, training, and information technology.

The sixth key concept of care coordination was defined as ‘need for coordination’. Four theoretical frameworks addressed the perceived or evaluated need for coordination. These frameworks focused on the importance of the need to exchange information and/or the need to provide and coordinate care.

The seventh key concept of care coordination was defined as the ‘administrative operational processes’. Six frameworks focused on processes to standardize or adapt care. Three kinds of methods of administrative operational processes were identified, each involving a different degree of feedback. These methods included:

- impersonal methods, involving standardized arrangements and minimal feedback, like guidelines
- personal methods, involving personal interactions between individual collaborators, or between a team and an assigned coordinator, with a considerable degree of feedback, like a personal contact between healthcare professionals
- group methods, involving joint planning and decision-making, with maximum feedback, like team meetings.

The eight key concept of care coordination was defined as ‘the exchange of information’. Nine theoretical frameworks addressed the transfer of information, ideas and opinions among the members of a team, within an organization or between organizations.

The ninth key concept of care coordination was defined as ‘goals’. Six theoretical frameworks considered the importance of setting common goals, sharing these goals and assuring collective ownership of these goals.

The tenth key concept of care coordination was defined as ‘roles’. Four theoretical frameworks focused on the definition of roles and the awareness of each other’s roles.

The eleventh key concept of care coordination was defined as the ‘quality of relationships’. The four theoretical frameworks addressing the quality of relationships promoted mutual respect and high quality collaboration.

These last four key concepts address the operational level. Key concepts at the operational level became increasingly important as the degree of task integration increased. Care coordination requires sufficient exchange of information, flexibility in defining new professional activities and roles and qualitative relationships.

Finally, the last three key concepts of care coordination emphasized the outcome. The twelfth key concept focused on ‘patient outcome’. Seven theoretical frameworks address patient outcome including the patient’s perception or the patient’s evaluation of healthcare professional performance regarding patient health status, patient satisfaction, the continuity of care, patient safety, efficiency, efficacy, availability, accessibility, and compatibility.

The thirteenth key concept of care coordination was defined as ‘team outcome’. Five theoretical frameworks included team behaviour and team satisfaction.

Lastly, the fourteenth key concept of care coordination focused on the ‘organizational or inter-organizational outcome’. Four theoretical frameworks incorporated outcomes like comprehensiveness, accessibility, compatibility, conflict, and efficiency of the organization. Here, we considered patient outcome to be the ultimate outcome of care coordination.

## Discussion

The present literature review identified seven theoretical frameworks for the study of care coordination that are currently applied in health care. These seven frameworks had not been described in the previous review of the Agency for Healthcare Research and Quality [5]. All of these seven theoretical frameworks, except one, were developed in the US. This gives rise to the question of whether these theoretical frameworks would be applicable to other healthcare systems.

Our search for theoretical frameworks was limited to the period of 2007–2010, because our aim was to update the 2007 review of the Agency for Healthcare Research and Quality. Four of the seven theoretical frameworks identified had been published before 2007, but were not included in the Agency for Healthcare Research and Quality review, because that review was only exploratory.

Our main focus, the in-depth analysis of both newly retrieved theoretical frameworks and theoretical frameworks identified in the Agency for Healthcare Research and Quality review, resulted in the identification of fourteen key concepts. These key concepts will facilitate the selection of a useful theoretical framework for developing, studying, and evaluating coordination strategies.

Two theoretical frameworks are certainly interesting as they included nearly all the identified key concepts, with the exception of external factors, cultural factors, and team outcome. These frameworks were 1) the Relational Coordination Theory, for exploring care coordination within an organization, and 2) the Multi-level Framework, which is an elaboration of the relational coordination theory that can be used to study care coordination between organizations. The Relational Coordination Theory relates the structure of an organization to the development of networks resulting in a certain outcome [12, 30]. The Multi-level Framework states that by using the same organizational mechanisms, both within and between organizations, networks are even more strengthened, thus resulting in more quality and efficiency of care [13]. These frameworks are also frequently applied in healthcare settings.

The most frequently cited theoretical framework was the Time, Interaction, and Performance Theory. This theory contains statements about the nature of groups, the behaviour of groups, and group processes [25]. Most of these references focused on teamwork in general, team processes, virtual teams and team’s use of technical tools, like Information and Communication Technologies to coordinate care.

The most common key concept in the existing theoretical frameworks for the study of care coordination was the exchange of information. This reflects a deficit in communication and information transfer [31]. Initiatives should be taken to support (electronic) data-sharing among primary caregivers and between primary and hospital caregivers to improve care coordination.

**Figure 2.**
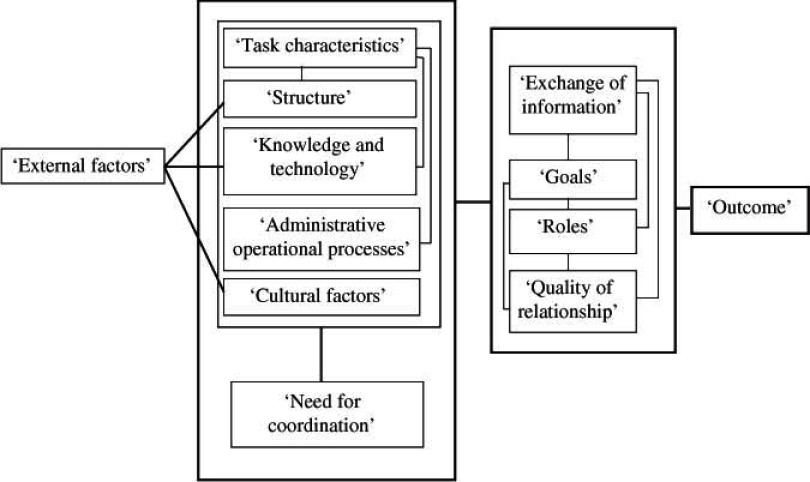
Relationships between key concepts of care coordination identified in the theoretical frameworks.

The following relationships between key concepts were identified in one or more of the retrieved theoretical frameworks: ‘External factors’ were linked with ‘structure’, ‘knowledge and technology’ and ‘cultural factors’. ‘Task characteristics’ related with ‘structure’, ‘knowledge and technology’ and ‘administrative operational processes’. The ‘need for coordination’ is influenced by ‘task characteristics’, ‘structure’, ‘knowledge and technology’, ‘administrative operational processes’ or ‘cultural factors’. These key concepts were also linked with ‘exchange of information’, ‘goals’, ‘roles’ and ‘quality of relationship’, resulting in a certain patient, team or (inter)organizational outcome. Finally ‘exchange of information’, ‘goals’, ‘roles’ and ‘quality of relationship’ relate to each other. These identified relationships are presented in Figure 2.

Coordination strategies must be adapted to the complexity of the situation. The existing networks between healthcare professionals become more important with increasing complexity and uncertainty of the ‘tasks’ [12, 30]. A study on the impact of several organizational mechanisms (relating to our identified key concepts ‘structure’, ‘knowledge and technology’ and ‘administrative operational processes’) found that a liaison function, team meetings, shared responsibility, and flexible work roles strengthened the relational coordination. The concept of ‘relational coordination’ emphasizes the importance of frequent, timely, accurately, and problem-solving effectiveness of ‘information exchange’, ‘goal’ sharing, ‘role’ recognition, and ‘quality of relationship’. Other organizational mechanisms, like information technology and routines, reduce the relational coordination having a negative impact on the outcomes when an unexpected event occurs. Although ‘care pathways’ are considered a type of routine, they can stimulate relational coordination, because they provide task agreements and in addition, they give insight into the care process as a whole, the role of each person, and the importance of the task the individual is expected to perform [12]. More research is required to determine which coordination strategy would be most effective for a given circumstance.

Coordination is related with other terms like integration. Integration also lacks clarity due to no shared definition and theoretical framework. Efforts are made to develop a model for integrated care [32, 33]. The identified elements for integration are relatively similar with the concepts identified for care coordination. The focus sometimes differs due to another ordering of the identified elements into clusters or key concepts, e.g., the elements of the cluster ‘patient-centeredness’ [33] are included in the following key concepts of care coordination: ‘roles’, ‘exchange of information’, ‘administrative operational processes’, ‘structure’ and ‘knowledge and technology’.

## Conclusion

The 14 interrelated key concepts of care coordination provide a base to develop or choose a framework for developing, studying, and evaluating coordination strategies. The relational coordination theory and the multi-level framework are interesting for studying care coordination within or between organizations as they are the most comprehensive.

## Reviewers

**Jean Macq**, MD, MPH, PhD, Professor at the Catholic University of Louvain, Belgium

**Henk Nies**, Professor, Dr, Chief Executive Officer, Vilans, Utrecht, Professor of Organisaion and Policy in Long-term Care, VU University of Amsterdam

**Dr. Susanne Kümpers**, Professor for Qualitative Health Research, Social Health Inequalities and Public Health Strategies Department of Nursing and Health Sciences, Fulda University of Applied Sciences

## Figures and Tables

**Table 1.** Overview of the newly retrieved theoretical frameworks for the study of care coordination

Name of theoretical framework, year of publication [ref]	Research agency	Purpose	Level of analysis	Application	
				Initial search (n)	Cited in Web of Knowledge (n)
Five phases of team coordination, 2001 [23]	Klein Associates Inc.	Define the characteristics and outcomes of team interactions/describe the features of team coordination	Team	1	0
Interaction Model, 2000 [24]	University of Michigan, Mental Research Institute	Identify five axioms of interactional communication	Team	1	0
Time, Interaction, and Performance Theory, 1991 [25]	University of Illinois, Psychology Department	Define the nature, behaviour, and processes of groups	Team	1	287
Interorganisational Network Theory, 1993 [26]	University of Maryland, Department of Sociology	Develop inter-organizational networks	Inter-organization	2	0
Cognitive Workflow Model, 2007 [27]	Colombia University, Department of Biomedical Informatics, Laboratory of Decision-Making and Cognition	Represent an intricate workflow applicable to all healthcare settings	Team	1	26
Framework of team performance, 2009 [28]	University of Aberdeen, School of Psychology	Determine the relationship between teamwork and patient or staff related outcomes	Team	1	25
Integrative model, 2009 [29]	University of Missouri, Department of Family and Community Medicine	Integrate an interdisciplinary team model and a model of successful collaboration	Team	1	2

**Table 2.**
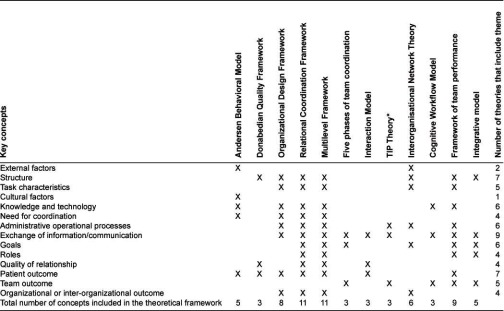
Synthesis of key concepts addressed in theoretical frameworks for the study of care coordination

*TIP theory=Time, Interaction, and Performance theory.
